# Use of remote sensing for linkage mapping and genomic prediction for common rust resistance in maize

**DOI:** 10.1016/j.fcr.2024.109281

**Published:** 2024-03-15

**Authors:** Alexander Loladze, Francelino A. Rodrigues, Cesar D. Petroli, Carlos Muñoz-Zavala, Sergio Naranjo, Felix San Vicente, Bruno Gerard, Osval A. Montesinos-Lopez, Jose Crossa, Johannes W.R. Martini

**Affiliations:** a International Maize and Wheat Improvement Center – CIMMYT, Mexico; b College of Agriculture and Environmental Sciences (CAES), University Mohammed VI Polytechnic (UM6P), Ben Guerir, Morocco; c Facultad de Telemática, Universidad de Colima, Colima, Mexico

**Keywords:** Remote sensing, Common rust, Vegetation Indices, Rp1

## Abstract

Breeding for disease resistance is a central component of strategies implemented to mitigate biotic stress impacts on crop yield. Conventionally, genotypes of a plant population are evaluated through a labor-intensive process of assigning visual scores (VS) of susceptibility (or resistance) by specifically trained staff, which limits manageable volumes and repeatability of evaluation trials. Remote sensing (RS) tools have the potential to streamline phenotyping processes and to deliver more standardized results at higher through-put. Here, we use a two-year evaluation trial of three newly developed biparental populations of maize doubled haploid lines (DH) to compare the results of genomic analyses of resistance to common rust (CR) when phenotyping is either based on conventional VS or on RS-derived (vegetation) indices. As a general observation, for each population × year combination, the broad sense heritability of VS was greater than or very close to the maximum heritability across all RS indices. Moreover, results of linkage mapping as well as of genomic prediction (GP), suggest that VS data was of a higher quality, indicated by higher $-\log\left(p\right)$ values in the linkage studies and higher predictive abilities for genomic prediction. Nevertheless, despite the qualitative differences between the phenotyping methods, each successfully identified the same genomic region on chromosome 10 as being associated with disease resistance. This region is likely related to the known CR resistance locus *Rp1*. Our results indicate that RS technology can be used to streamline genetic evaluation processes for foliar disease resistance in maize. In particular, RS can potentially reduce costs of phenotypic evaluations and increase trialing capacities.

## Highlights

• Remote sensing (RS) allows for high-throughput phenotyping in genetic evaluations.
• RS and visual scores (VS) from trained staff identified the same genomic regions.
• Data quality of VS was higher than that of vegetation indices obtained from RS.
• An index more specific to disease symptoms may improve data quality further.

## 1 Introduction

Remote sensing (RS) is currently a central topic of investigation in high-throughput phenotyping and precision agriculture (Tsouros et al., 2019). Images from unmanned aerial vehicles (UAVs) and satellites have been used to estimate a leaf area index and yield of rice (Duan et al., 2021, Gong et al., 2021), to estimate a wheat harvest index (Campoy et al., 2020), to predict the optimal harvest date for maize (Xu et al., 2019), and to monitor maize lodging (Tirado et al., 2021) or fertilizer response (Schut et al., 2018). Moreover, several authors addressed the application of RS for disease detection in crop production. For instance, RS has been proposed for the detection and mapping of different diseases in sugar beet (Hillnhütter et al., 2011) and for early disease detection of red leaf blotch disease in almond trees (López-López et al., 2016). Likewise, Kerkech et al. (2018) identified infected areas of grapevines with UAV images, while Zarco-Tejada et al. (2018) showed that infections by a bacterial pathogen *Xylella fastidiosa* could be detected with RS before symptoms were visible in an olive orchard at a landscape scale.

Recently, RS has also gained more attention for applications in plant pathology and resistance breeding focusing on the discovery of major resistance genes or on the selection of genotypes with “quantitative” resistance. Compared to the application of RS for disease detection in a production field, a more refined quantification of symptom severity is necessary to be used for resistance breeding. Switching from conventional phenotyping with a visual score (VS) to RS can potentially accelerate the evaluation process and allow a higher number of plots to be evaluated, thus enabling high-throughput phenotyping systems. In addition, RS could help to improve the quality of phenotyping by reducing human errors, especially if the personnel is inexperienced or the scorings between different teams are not fully aligned.

So far, the accuracy assessment of RS for scoring plant disease resistance has mainly focused on the similarity of RS data and human-generated VS, mostly considering the VS as the benchmark. Loladze et al. (2019) used image data provided by an UAV for the quantification of the severity of Tar Spot Complex, a fungal foliar disease of maize. The authors found that the phenotypic data obtained through RS was very similar to conventional VS phenotyping. Likewise, in another study, the VS for disease severity of *Ascochyta* blight in Chickpeas was shown to be well correlated with RS data (Zhang et al., 2019).

In breeding programs, phenotypic data is used for the genomic characterization of genetically heterogeneous germplasm. Therefore, the question arises how results of follow-up genomic analyses compare when based on the different types of phenotypic data. For instance, RS and VS data can be compared based on the similarity of detected peaks and the size of p-values obtained from linkage mapping. Assuming that RS data is more precise, as it eliminates the subjective human factor, the p-values related to genomic regions associated with disease resistance should be smaller for RS data than when using VS. Similarly, one can speculate that the predictive ability of genomic prediction should increase when using RS traits as the dependent variable.

Common rust (CR) in maize (*Zea mays L.*), caused by an obligate fungal pathogen *Puccinia sorghi* Schwein, is one of the most pervasive diseases in many maize-growing regions (Sserumaga et al., 2020), and yield losses of up to 49% were reported in susceptible germplasm (Groth et al., 1983). Because of the severity of the disease, several authors have investigated the genetic basis of resistance to CR and have identified different quantitative trait loci (QTL) (Kerns et al., 1999, Danson et al., 2008, Zheng et al., 2018, Ren et al., 2021).

In this study, we investigated how the results of genomic analyses compare when the phenotypic data is derived from RS (UAV-derived multispectral and thermal infrared image data) as opposed to when conventional VS data is used. We considered different (vegetation) indices obtained from UAV images as RS data and compared the results of follow-up linkage mapping and genomic-enabled prediction (GP) analyses to the results from the same analyses based on VS data. In particular, we addressed the hypothesis that a “potentially increased objectivity” of RS will lead to higher data quality and thus will improve results.

RS and VS data were obtained from two-year evaluations of three newly generated F2-based doubled haploid (DH) populations of 320, 260 and 320 lines, respectively.

## 2 Materials and Methods

### 2.1 Development of plant material

Three different maize genotypes adapted to the high plains of Mexico (CHWTI23, CHWTI59, and DTMA-17) were chosen for the study as potential donors with resistance to CR. The genotypes were selected based on their high resistance levels to the disease observed over several years and various trials at CIMMYT’s El Batan (State of Mexico) experimental station (data not shown). Each resistant genotype was crossed with the CR-susceptible parent DTMA-85 at CIMMYT’s Agua Fria Experimental Station (Puebla, Mexico) in spring 2017. The F1 generation was planted the following season in the fall of 2017 at El Batan, and for each cross, 1000 F2 seeds were transferred to CIMMYT’s Doubled Haploid (DH) program. The first generation of DH lines was obtained in the Fall of 2018 and was planted for seed increase at the Agua Fria station.

### 2.2 Experimental design for evaluation of CR symptoms

The obtained DH entries were evaluated for CR resistance at the El Batan experimental station. The resistant and susceptible parental lines were also included in the trials. The number of evaluated entries were 320, 260 and 320 for the crosses CHWTI23/DTMA-85 (“population 1″), CHWTI59/DTMA-85 (“population 2″) and DTMA-17/DTMA-85 (“population 3″), respectively. In total, the three distinct populations were planted over two cropping seasons, 2019 and 2020, in trials with two replications of each genotype in a lattice design. Each field plot consisted of two 2.5 m long rows with a distance of 0.75 m between the rows and a plant spacing of 0.25 m within each row.

### 2.3 Inoculation and visual scoring

The inoculations were conducted by spraying a water-Tween 20 suspension of *P. sorghi* urediniospores over two consecutive days, approximately one month before flowering. The urediniospores were obtained from a spore collection from a previous year that were desiccated and stored at − 20° C. Spore concentration was only visually “adjusted” to a “dark brown” color. The concentration of spores was not quantified in detail.

The evaluations were conducted by trained personnel starting after the appearance of first symptoms, usually during silking. The symptoms were visually evaluated at three time points based on a 1 to 9 scale reflecting the severity of symptoms (1 =very resistant, 9 = very susceptible). The first time point was -as mentioned before- after the first appearance of symptoms. The second time point was approximately two weeks after the first time point, and the third time point was two weeks after the second. For this study, only the data of the third time point was used.

### 2.4 Image acquisition and processing

A fixed-wing UAV-based remote sensing eBee Plus platform was used (SenseFly Ltd., Cheseaux-Lausanne, Switzerland), as described by Loladze et al. (2019). A multispectral Parrot Sequoia camera (Parrot Drone SAS, Paris, France) captured the following wavelengths: 550 nm (40 nm full width at half maximum, FWHM), 660 nm (40 nm FWHM), 735 nm (10 nm FWHM), 790 nm (40 nm FWHM). Additionally, in separate flights, the UAV was equipped with a thermal infrared camera, ThermoMAP (7.5–13.5 µm, Airinov, Paris, France). Flights were conducted at a height of 55 m above ground, around noon under sunny conditions. The images were acquired with 80% lateral and 80% longitudinal overlaps, with a ground resolution of 6 and 12 cm for the multispectral and thermal cameras, respectively. The multispectral camera was radiometrically calibrated before each flight based on the standard camera panel provided by the manufacturer. Additionally, the incident light sensor of the multispectral camera measured sun irradiance for radiometric adjustment of images taken under varying light conditions. Three flights were performed in each crop cycle (2019, 2020) on the same days as the VS evaluations were conducted, or at most one day before or after. Images were geotagged for subsequential orthomosaic processing with Pix4D Mapper® (v3.3.24; Pix4D, Lausanne, Switzerland) and then converted into reflectance or surface temperature data, depending on the respective camera (multispectral and thermal infrared).

**Fig. 1:**
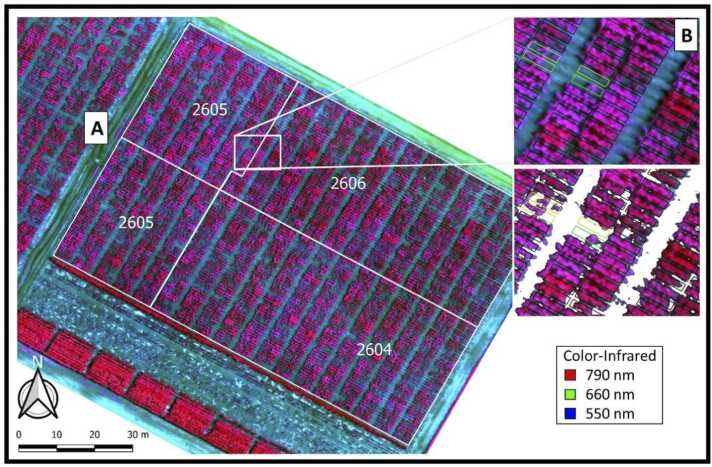
Location of the experiment at the International Maize and Wheat Improvement Center (CIMMYT), El Batan experimental station in Mexico. Color-infrared image (790, 660, 550 nm) of the experimental nurseries (A) and derived crop-mask used prior within-plot data extraction (B). Trial number 2604, 2605, and 2606 correspond here to populations 1, 2 and 3, respectively.

A vegetation mask was derived from an offset of the soil line through a red *vs* near-infrared (NIR) scatter plot from the first acquired orthomosaic of each experimental year before the extraction of orthomosaic data. The line joining the darker and brighter soil points defined the soil line, which was further adjusted by a correction factor and then applied to all orthomosaics, masking within-plot background pixels (Fig. 1B). The vegetation-masked orthomosaics were then used for within-plot data extraction. The within-plot masked pixels were selected and averaged using Python (python 3.5.6, package rasterstats 0.13.1). A minimum of 1/10 of within-plot effective pixels, i.e. approximately 30 pure pixels, was considered as a threshold to identify missing plots.

In total, ten different vegetation indices (VIs) were calculated for each orthomosaic (Table 1). For each wavelength required to calculate the VIs, the closest wavelength response of the multispectral signal (consisting of 550, 660, 735, 790 nm) was considered, taking into account the FWHM of each channel. In addition, the canopy temperature was extracted from the thermal imagery. As for the VS, only the third time point was used in this study.

**Table 1:** Overview of the different vegetation indices used in this work.

Vegetation Index	Equation	Reference
	**Structural Indices**	
Normalized Difference Vegetation Index (NDVI)	$\frac{R_{800}-R_{670}}{R_{800}+R_{670}}$	Rouse et al. (1973)
Renormalized DVI (RDVI)	$\frac{R_{800}-R_{670}}{\sqrt{R_{800}{+R}_{670}}}$	Roujean and Breon (1995)
Modified Simple Ratio (MSR)	$\frac{R_{800}}{R_{670}}-{\left(\frac{R_{800}}{R_{670}}\right)}^{-0.5}+1$	Chen (1996)
Optimized Soil-Adjusted Vegetation Index (OSAVI)	$\frac{{\left(1.16\right)*(R}_{800}-R_{670})}{R_{800}+R_{670}+0.16}$	Rondeaux et al. (1996)
Modified Chlorophyll Absorption in Reflectance Index (MCARI_1_)	$1.2*[2.5*\left(R_{800}-R_{670}\right)-1.3*\left(R_{800}-R_{550}\right)]$	Haboudane et al. (2004)
Modified Chlorophyll Absorption in Reflectance Index (MCARI_2_)	$\frac{1.2*[2.5*\left(R_{800}-R_{670}\right)-1.3*\left(R_{800}-R_{550}\right)]}{\sqrt{{\left(2*R_{800}+1\right)}^{2}-\left(6*R_{800}-5*\sqrt{R_{680}}\right)-0.5}}$	Haboudane et al. (2004)
	**Chlorophyll Indices**	
Triangular Vegetation Index (TVI)	$0.5*[120*\left(R_{750}-R_{550}\right)-200*\left(R_{670}-R_{550}\right)]$	Broge and Leblanc (2001)
Gitelson and Merzlyak (GM1)	$\frac{R_{750}}{R_{550}}$	Gitelson and Merzlyak (1997)
Pigment specific simple ratio for chlorophyll a (PSSRa)	$\frac{R_{800}}{R_{680}}$	Blackburn (1998)
	**RGB Indices**	
G	$\frac{R_{550}}{R_{670}}$	Zarco-Tejada et al. (2005)

### 2.5 Phenotypic adjustment

For each combination of population and year, a linear mixed model was used to derive a best linear unbiased estimate (BLUEs) of each trait (reflection at a given wavelength, vegetation indices, visual score) for each genotype of each bi-parental population in each year. The linear mixed model was defined as

(1) y=μ+g+block+ϵ

Here, y denotes the measured phenotypes, μ denotes a fixed population effect and g the genotypic effect which was also modeled as being fixed. Moreover, block and ϵ where random effects of the block and of the error of the specific plot on which the phenotype was measured.

### 2.6 Broad-sense heritability for each population × year combination

For the estimation of broad-sense heritability for each population × year combination, the same model was used but g was modeled as a random effect to obtain an estimate of the genetic variance $\sigma_{g}^{2}$. Broad sense heritability was estimated as

$$H^{2}=\frac{\sigma_{g}^{2}}{\sigma_{g}^{2}+\frac{1}{2}\sigma_{\epsilon }^{2}}$$

Here, $\sigma_{\epsilon }^{2}$ is the error variance and the factor $\frac{1}{2}$ reflects the 2 replications of each genotype. All mixed model analyses were performed based on ASREML-R (Butler et al., 2009). Broad-sense heritability characterizes the accuracy (content of information) of the adjusted phenotypes obtained from Eq. (1).

### 2.7 Genotypic data

Leaf tissue from one plant per DH line was sampled and frozen at − 80 °C, which was followed by lyophilization and DNA extraction according to a modified CTAB method (CIMMYT, Laboratory protocols, 2005). The obtained DNA was quantified, diluted to equal concentration (100 ng/ul), and genotyped by the Genetic Analyses Service for Agriculture (Spanish acronym SAGA) established at CIMMYT, El Batan, Mexico.

To develop the genomic profile of each sample, SAGA uses the DArTseq™ method, created by Diversity Arrays Technology (DArT) (Sansaloni et al., 2011), a high-throughput genotyping method with the reduction of the genome complexity as the first and most important step. This obtains a genomic representation of each sample by digestion of the DNA using a combination of two restriction enzymes, *Pst*I (CTGCAG) and *Hpa*II (CCGG). A multiplexing system in a single assay uses barcode sequences, which identify each DNA fragment as belonging to a specific sample on the DNA plate. For each 96 well plate, almost 18% of the samples were replicated to improve reproducibility and robustness of the data. After the PCR process, equimolar amounts of amplification products from each sample were pooled per plate, followed by fragment sequencing on an Illumina Novaseq 6000 equipment. A proprietary analytical pipeline developed by DArT P/L was used to generate allele calls for presence/absence variation (Silico-DArT) and single nucleotide polymorphism (SNP) markers (Petroli and Kilian, 2019). Only SNP markers were used here, and the data was filtered using parameters such as Call Rate and Reproducibility. Markers have been mapped to the *Zea mays* L. reference genome (B73, V.4). After discarding markers with more than 40% missing values or a minor allele frequency lower than 0.02, 9051 markers remained for further analysis. We used a high threshold of 40% of missing values, since we treated the data of three different populations together and wanted to avoid discarding markers for not being present in one of the populations. The remaining missing data was “imputed” by the mean of the available data for the respective marker.

### 2.8 Linkage mapping

For linkage mapping, the R (R Core Team, 2020) package RRBLUP (Endelman, 2011) was used. The function GWAS() was applied to the marker data, together with the adjusted phenotypic data (VS and RS). The function performs genome-wide linkage analyses based on a mixed model with fixed and random effects (Yu et al., 2006). We neither specified fixed effects, nor specified a kinship matrix. If the kinship matrix is not specified, it is calculated automatically from the markers to capture polygenic effects.

### 2.9 Genomic prediction based on genomic best linear unbiased prediction (GBLUP)

The adjusted phenotypes were used together with the genotypic data to test whether the predictive ability was comparable between RS and VS data. For each population × year combination, 10% of the adjusted phenotypes were removed (“test set”) and predicted by the remaining 90% (“training set”). This was repeated 100 times and the average Pearson correlation between adjusted phenotypes and their predictions (for the test set) as well as the corresponding standard errors were measured.

The model for genomic prediction was:

y=μ+g+ϵ

with y the adjusted values (see “Phenotypic adjustment”), μ a fixed effect, $g∼N(0,\sigma_{g}^{2}G)$ and G the genomic relationship matrix defined as $G=XX^{t}/9051$ with X the n×9051 matrix giving the marker states of the n individuals (and 9051 being the number of markers). Moreover, $\epsilon ∼N(0,\sigma_{\epsilon }^{2}I)$ with I the identity matrix, which means that the error terms are independent and identically distributed.

Variance components were estimated using the R package regress (Clifford and McCullagh, 2006, Clifford and McCullagh, 2020) and the prediction was based on the mixed model equations (Henderson, 1973) with the estimated variance components. Regress() uses by default a restricted log likelihood approach and the Newton-Raphson algorithm to locate the maximum of the log-likelihood.

### 2.10 Data availability

We recorded RS traits at three different time points for each of the six population × year combinations. We considered the data of each time point separately and also calculated the area under the disease progression curve (AUDPC; Vanderplank, 1963), which is a weighted mean of the values at the three time points. Since the variance in the RS traits was highest at time point 3 (TP3), the AUDPC had a very high correlation to the data of TP3, and the heritabilities of RS traits tended to be highest at TP3 (except for the MSR index, data not shown), we decided to base the presentation of our results on the third evaluation only.

The corresponding data can be found on CIMMYT’s dataverse repository (https://data.cimmyt.org/dataset.xhtml?persistentId=hdl:11529/10548898). For more information on the data structure see also Loladze et al. (2023).

## 3 Results

### 3.1 Broad sense heritability, correlation and across-year correlations of visual score (VS) and indices

#### 3.1.1 Broad sense heritabilities of individual population × year combinations

Broad sense heritabilities characterize the information content of the adjusted phenotypes. Estimated (broad sense) heritabilities are summarized in Table 2. For five of the six combinations of population (1, 2, 3) by year (2019, 2020), the estimated heritability of VS was the highest across all traits. Only for population 3, 2020, an RS trait -the RGB index G- showed a higher heritability than the VS. Furthermore, while none of the indices reached a heritability of 0.9 or higher in 2019, TVI, G, PSSRa, NDVI, RDVI, OSAVI and MCARI1 and MCARI2, all reached an estimated heritability of 0.94 or higher for populations 2 and 3 in 2020. The difference in heritabilities of the RS traits across the two years may be related to differences in the disease progression or growing conditions. The light conditions when the images were taken should not be the most relevant factor, since the camera is radiometric calibrated before each flight.

**Table 2:** Estimated (broad-sense) heritabilities of all traits and individual population × year combinations. The highest values of each row are highlighted in bold.

	VS	“Raw” Spectral Data					Indices									
		thr	gre	red	nir	reg	TVI	GM1	G	PSSRa	NDVI	RDVI	OSAVI	MSR	MCARI1	MCARI2
Pop 1, 2019	**0.94**	0.74	0.66	0.71	0.86	0.82	0.87	0.83	0.84	0.85	0.84	0.86	0.86	0.49	0.87	0.86
Pop 2, 2019	**0.94**	0.67	0.47	0.65	0.87	0.83	0.89	0.83	0.86	0.83	0.85	0.87	0.87	0.32	0.88	0.88
Pop 3, 2019	**0.94**	0.55	0.49	0.66	0.84	0.80	0.86	0.79	0.83	0.82	0.82	0.85	0.84	0.36	0.86	0.85
Pop 1, 2020	**0.95**	0.59	0.91	0.91	0.86	0.86	0.86	0.81	0.90	0.89	0.89	0.87	0.88	0.90	0.87	0.88
Pop 2, 2020	**0.95**	0.76	0.90	0.92	0.92	0.89	0.94	0.90	**0.95**	0.94	**0.95**	**0.95**	**0.95**	0.85	0.94	**0.95**
Pop 3, 2020	0.95	0.73	0.90	0.93	0.92	0.91	0.94	0.92	**0.96**	0.95	0.95	0.95	0.95	0.88	0.94	0.95

It is noteworthy that heritabilities of disease-related traits influenced by a small number of genes with strong effects are expected to be high in DH populations resulting from contrasting parents and a consequently relatively high genetic variance (due to the expected allele frequency of 0.5). Moreover, note that the considered broad-sense heritabilities characterize the data quality of the adjusted phenotypes obtained from each population × year combination individually, which in particular means for the fixed environment. The reported “broad sense with fixed environment” heritabilities do not capture genotype × environment variation. These “broad-sense” heritabilities allow us to interpret a potential lack of genetic signal in linkage mapping as rather being a result of low data quality (“trait is not measured well or highly variable due to external influences”) or as rather being a result of the trait not capturing the disease symptoms well.

#### 3.1.2 Correlation of indices and VS at the third evaluation

For population 1 in 2019, the indices G, TVI, MCARI1 and MCARI2 showed the highest absolute correlations to VS (−0.69, −0.66, −0.65 and 0.65, respectively). In general, one would aim at a high absolute correlation, that is a maximum if the correlation is positive, or a minimum if the correlation is negative. Correlations between indices and VS were mostly negative, but we may refer to their absolute value in the following.

For the same population in 2020, the highest absolute correlation with VS were 0.73, 0.68 and 0.68 for G, NDVI and PSSRa, respectively.

For population 2 in 2019, NDVI, MCARI1, MCARI2, OSAVI, RDVI and TVI showed the highest absolute correlation to VS data (0.74 for NDVI and 0.73 for each of the other indices). The indices G and PSSRa exhibited a correlation of − 0.72 with VS. For population 2 in 2020, the indices with highest absolute correlation with VS were G (with a correlation of −0.86), PSSRa (−0.84) and OSAVI (−0.83) and MCARI2 (−0.83).

For population 3 in 2019, G (−0.73), MCARI1 (−0.72) and TVI (−0.72) had the highest absolute correlation with the VS data. For 2020, G (−0.87), PSSRa (−0.85) and NDVI (−0.82) had the strongest correlation with VS data.

Overall, in five of the six population × year combinations, G had the highest correlation with VS. The only exception was population 2 in 2019 which showed a correlation coefficient of − 0.72 (slightly higher than the correlation of −0.74 of NDVI and VS).

#### 3.1.3 Across-year correlation of traits

A certain “stability” of the VS data has been illustrated by the high across-year correlation of the trait for each population (Table 3), which could be attributed to the higher heritability and potentially to a smaller genotype × year interaction for the VS data.

**Table 3:** Across year correlation of each trait for the three populations. The highest values of each row are in bold.

	**VS**	**Thr**	**Gre**	**Red**	**nir**	**reg**	**TVI**	**GM1**	**G**	**PSSRa**	**NDVI**	**RDVI**	**OSAVI**	**MSR**	**MCARI1**	**MCARI2**
**Population 1**	**0.71**	0.39	0.44	0.40	0.62	0.58	0.62	0.57	0.57	0.58	0.59	0.64	0.63	0.27	0.64	0.64
**Population 2**	**0.80**	0.43	0.42	0.51	0.73	0.65	0.71	0.67	0.63	0.67	0.68	0.73	0.72	0.35	0.73	0.72
**Population 3**	**0.75**	0.34	0.27	0.44	0.64	0.57	0.64	0.54	0.58	0.57	0.60	0.64	0.63	0.20	0.66	0.64

### 3.2 Comparison of linkage mapping results when based on VS data or RS indices

**Fig. 2:**
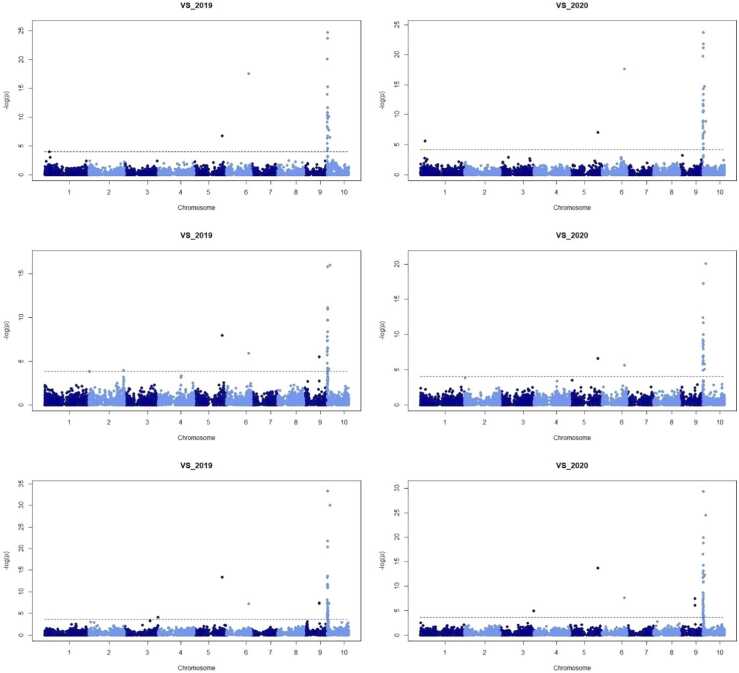
Manhattan plots of the linkage analyses for the 6 population × year combinations and the **visual score (VS)** as trait. The 1st row shows the $-\log\left(p\right)$ values of the linkage analysis based on population 1, the 2nd row shows the results of population 2, and the 3rd row presents the results for population 3. The dashed line in the Manhattan plot indicates a threshold for a false discovery rate (FDR) of 0.05.

The results of linkage mapping when using the VS data as phenotype are shown in Fig. 2. The dashed line in the Manhattan plot represents a threshold for a false discovery rate (FDR; Benjamini and Hochberg, 1995) of 0.05, based on the qvalue package (Storey and Tibshirani, 2003, Endelman, 2011).

Considering the results based on the VS, a genomic region associated with CR resistance was discovered on Chromosome 10 for all three populations. Position 2954,643 bp showed the greatest −log(p) value(s) for population 1 and 3, with values of 24.77 and 33.32 in 2019 respectively, and 23.69 and 29.33 in 2020, respectively (see Fig. 2 and Table 4). In population 2, the position 20,858,205 bp showed the highest −log(p) value in both years (15.98 and 20.10 in 2019 and 2020, respectively). This region between 2 and 21 Mbp was consistently identified in all three populations and in each year when using the VS data. Note here that the markers cover around 150 Mbp for chromosome 10, which means that the distance between 2 and 21 Mbp seems small in the Manhattan plots of Fig. 2.

**Table 4:** Position of the marker with greatest −log(p) value in a linkage analysis based on each of the traits. Whenever a marker on chromosome 10 showed a −log(p) value larger than the threshold for an FDR of 0.05, the maximal $-\log\left(p\right)$ across all markers was found on chromosome 10. “Not detected” means that no marker on chromosome 10 exceeded the FDR threshold. The markers at positions 2954,643 and 20,858,205 were identified when using the VS and are highlighted in bold.

		Population 1		Population 2		Population 3	
		2019	2020	2019	2020	2019	2020
VS	Pos. −log(p)	**2954,643**	**2954,643**	**20,858,205**	**20,858,205**	**2954,643**	**2954,643**
		24.77	23.69	15.98	20.10	33.32	29.33
thr	Pos. −log(p)	Not detected	Not detected	Not detected	**20,858,205**	Not detected	**20,858,205**
					5.16		9.38
gre	Pos. −log(p)	Not detected	Not detected	Not detected	Not detected	Not detected	Not detected
red	Pos. −log(p)	Not detected	Not detected	**2954,643**	**20,858,205**	3878,003	**2954,643**
				9.20	6.33	9.76	14.16
nir	Pos. −log(p)	2368,199	Not detected	**20,858,205**	3909,507	**2954,643**	**20,858,205**
		10.93		12.80	6.55	14.569	12.07
reg	Pos. −log(p)	2368,199	Not detected	**20,858,205**	Not detected	**2954,643**	3909,507
		11.66		13.23		13.33	7.42
TVI	Pos. −log(p)	2368,199	Not detected	**2954,643**	**20,858,205**	**2954,643**	**2954,643**
		11.36		13.76	7.93	14.88	13.86
GM1	Pos. −log(p)	Not detected	Not detected	**2954,643**	3909,507	3878,003	**20,858,205**
				10.28	5.63	11.78	11.67
G	Pos. −log(p)	**2954,643**	2639,580	**2954,643**	**20,858,205**	**2954,643**	**2954,643**
		12.16	6.88	14.75	11.40	14.19	19.04
PSSRa	Pos. −log(p)	**2954,643**	Not detected	**2954,643**	**20,858,205**	**2954,643**	**2954,643**
		7.92		12.09	9.67	12.16	18.41
NDVI	Pos. −log(p)	2368,199	Not detected	**2954,643**	**20,858,205**	**2954,643**	**2954,643**
		7.97		12.00	7.77	13.59	16.40
RDVI	Pos. −log(p)	2368,199	Not detected	**2954,643**	**20,858,205**	**2954,643**	**2954,643**
		9.43		11.92	8.03	14.19	15.80
OSAVI	Pos. −log(p)	2368,199	Not detected	**2954,643**	**20,858,205**	**2954,643**	**2954,643**
		8.97		12.06	8.10	14.04	16.29
MSR	Pos. −log(p)	Not detected	Not detected	Not detected	Not detected	Not detected	Not detected
MCARI1	Pos. −log(p)	2368,199	Not detected	**2954,643**	**20,858,205**	**2954,643**	**2954,643**
		10.66		12.57	8.09	14.76	15.24
MCARI2	Pos. −log(p)	2368,199	2309,109	**2954,643**	**20,858,205**	**2954,643**	**2954,643**
		10.12	4.90	12.72	8.93	14.29	16.82

For each of the RS traits, we considered the region on chromosome 10 as being detected if a marker on chromosome 10 had a −log(p) value above the threshold of FDR 0.05. Whenever this region was detected, the maximal $-\log\left(p\right)$ value across all genomic markers was also found in this region. The highest $-\log\left(p\right)$ value and the corresponding marker for all population × year combinations and for all traits are summarized in Table 4.

When comparing the $-\log\left(p\right)$ values of the VS to the RS traits, we see that the VS led to the highest values across all traits for each population × year combination, suggesting that the VS data captured the symptoms of the disease better than the RS indices. In particular, this observation holds true for the cases in which the estimated heritability of an RS index was on the same level as the heritability of VS. For instance, the index G showed a slightly higher heritability than VS for population 3 in 2020, but the $-\log\left(p\right)$ value was smaller than for the VS (19.04 with G compared to 29.33 for VS). Therefore, G seems to be less specific to the disease symptoms than VS.

**Fig. 3:**
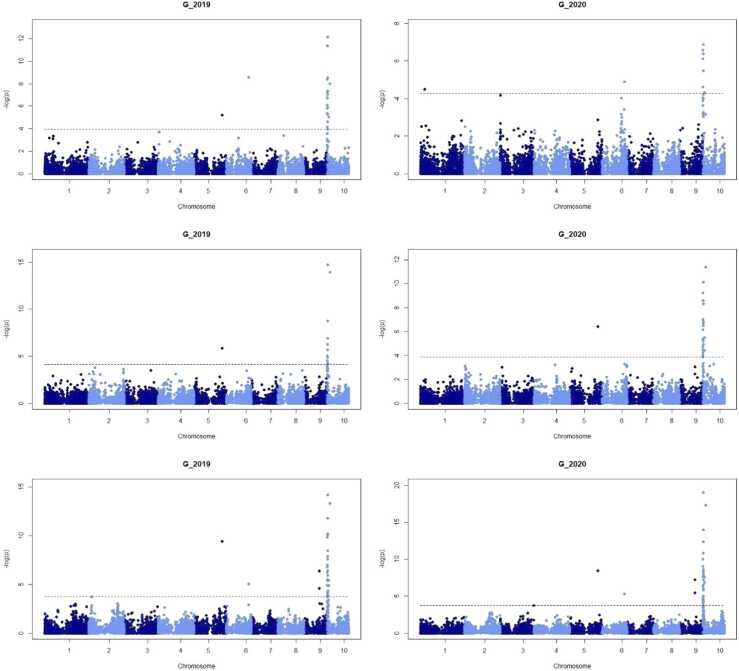
Manhattan plots of the linkage analyses for the 6 population × year combinations and the RS trait “G”. The 1st row shows the $-\log\left(p\right)$ values of the linkage analysis based on population 1, the 2nd row shows the results of population 2, and the 3rd row presents the results for population 3. The dashed line in the Manhattan plot indicates a threshold for a false discovery rate (FDR) of 0.05.

Considering the two markers showing the strongest signal for the trait VS, in five of the six combinations of population × year, G and PSSRa detected one of the two markers that were identified in the analyses based on the VS data (Table 4). Interestingly, for population 2 and 2019, both indices assigned the strongest signal to marker 2954,643, although the VS of this population × year combination pointed at position 20,858,205 bp. Moreover, besides the VS data, G had the largest $-\log\left(p\right)$ values in five of the six cases. The Manhattan plots of the linkage analyses based on G are presented in Fig. 3.

Comparing the linkage analyses for each trait × year combination across populations, the observed genetic signals captured with RS traits were, in general, weaker for population 1. In contrast, the VS found stronger signals for population 1 than for population 2 in each year. The latter is expected to some degree due to the smaller population size of population 2 (260 DH lines) compared to population 1 and 3 (320 DH lines each).

We also considered a joint linkage mapping analysis combining the three data sets. As expected -since the three populations already point at the same genomic region- the maximum $-\log\left(p\right)$ values were even higher reaching 27.33, 21.25, for the index G in 2019 and 2020 and 52.18, 46.89 for VS in 2019 and 2020. For the combined data set of the three populations, the marker at 2954,643 bp was confirmed, which seems expectable due to the two larger populations 1 and 3, both pointing at this position. It is likely that this is the most precise estimation of the position of the causative variant, but there could also be population specific causative alleles. In case that the causative allele of population 2 actually has a slightly different position, this would not be visible when combining the three data sets.

Please note that there were also other markers identified as being associated with the resistance which are not in the main region on chromosome 10 but on chromosomes 5, 6, and 9. The exact positions of the markers with strongest signal and when combing the three data sets were marker positions 194,463,126 bp on chromosome 5, 154,393,240 bp on chromosome 6, and 106,229,626 bp on chromosome 9, for the four cases of G and VS in 2019 and 2020. There are no clear peaks on chromosome 5 and chromosome 6, but the markers are relatively isolated (as also visible in Fig. 2, Fig. 3). This could suggest that the positions of the markers are not correctly mapped when using the reference genome B73, V.4. Contrarily, the region on chromosome 9 shows several markers with elevated $-\log\left(p\right)$ value (Population 3 in Fig. 2, Fig. 3).

### 3.3 Comparison of genomic prediction of VS data and RS indices

The predictive abilities, that is, the average Pearson correlation of the predicted values and the known phenotypes of the test set in a cross validation are summarized in Table 5. The VS data showed the highest predictive ability across traits for five of the six population × year combinations. Only for population 2 in 2020, the index GM1 reached a higher predictive ability of 0.66±0.01 compared to 0.65±0.01 for the VS, which was, however, within the interval of the standard error. When correlating the heritabilities (Table 2) to the predictive abilities (Table 5), the values between 0.72 and 0.95 were observed, indicating that the predictive ability may, to a larger extent, be driven by the heritability of the trait, or in other words, by the quality of the phenotypic data (Table 6).

**Table 5:** Predictive ability of genomic prediction for the different traits and for each population × year combination. The standard error is $\frac{\mathrm{sd}}{\sqrt{n}}$.

	Population 1		Population 2		Population 3	
	**2019**	**2020**	**2019**	**2020**	**2019**	**2020**
VS	0.78±0.01	0.72±0.01	0.74±0.01	0.65±0.01	0.63±0.01	0.55±0.01
thr	0.40±0.02	0.43±0.02	0.42±0.02	0.40±0.02	0.11±0.02	0.15±0.01
gre	0.42±0.02	0.57±0.01	0.29±0.02	0.52±0.01	0.33±0.01	0.33±0.02
red	0.48±0.01	0.59±0.01	0.57±0.01	0.56±0.01	0.47±0.01	0.45±0.01
nir	0.63±0.01	0.53±0.01	0.56±0.01	0.56±0.02	0.52±0.01	0.49±0.01
reg	0.62±0.01	0.51±0.01	0.50±0.01	0.48±0.02	0.49±0.01	0.46±0.01
TVI	0.63±0.01	0.52±0.01	0.63±0.01	0.51±0.02	0.54±0.01	0.50±0.01
GM1	0.53±0.01	0.51±0.01	0.60±0.01	0.66±0.01	0.50±0.01	0.45±0.01
G	0.62±0.01	0.60±0.01	0.66±0.01	0.52±0.01	0.52±0.01	0.52±0.01
PSSRa	0.58±0.01	0.59±0.01	0.64±0.01	0.60±0.01	0.49±0.01	0.51±0.01
NDVI	0.60±0.01	0.57±0.01	0.65±0.01	0.58±0.01	0.55±0.01	0.48±0.01
RDVI	0.62±0.01	0.55±0.01	0.64±0.01	0.59±0.01	0.54±0.01	0.51±0.01
OSAVI	0.61±0.01	0.56±0.01	0.65±0.01	0.58±0.01	0.55±0.01	0.50±0.01
MSR	0.29±0.02	0.57±0.01	0.22±0.02	0.49±0.01	0.30±0.02	0.40±0.01
MCARI1	0.63±0.01	0.54±0.01	0.63±0.01	0.57±0.01	0.54±0.01	0.51±0.01
MCARI2	0.62±0.01	0.56±0.01	0.64±0.01	0.58±0.01	0.53±0.01	0.52±0.01

**Table 6:** Correlation of heritability of Table 2 and predictive ability of Table 5 across population × year combinations.

Population 1		Population 2		Population 3	
**2019**	**2020**	**2019**	**2020**	**2019**	**2020**
0.94	0.80	0.93	0.72	0.85	0.95

Comparing the results of the linkage analyses to the predictive abilities, we see that the RS traits capture disease symptoms to different extents. Consider for instance the G and GM1 indices for population 2, 2020, for which G had a lower predictive ability than GM1 (0.52±0.01 compared to 0.66±0.01), but a much stronger signal in the linkage analysis ($-\log\left(p\right)$ values of 11.40 compared to 5.63).

## 4 Discussion

### 4.1 Genetic signal on chromosome 10

**Fig. 4:**
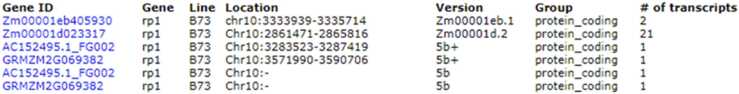
Gene model information on *Rp1* from MaizeGDB (Woodhouse et al., 2021).

The region on chromosome 10 is likely related to the *Rp1* cluster for which at least 14 CR race-specific resistance genes variation have been reported (Collins et al., 1999, Chavan et al., 2015). Lin et al. (2021) reported a location between 2.7 Mbp and 3.7 Mbp on chromosome 10 for the *Rp1* cluster for the B73 map and a window of 3.4 Mbp to 4.85 Mbp on the inbred line A188. We had used a B73 map and the position at 2954,643 bp, which we identified as the most relevant here, locates within the reported interval. This chromosomal region is recognized as being very complex and the distinction of allelic variants proved to be challenging (Quade et al., 2021). The detailed investigation of the allelic variation was not covered by the scope of the current research, but the allelic variation of the three different resistance donors used here may be a topic for future investigations. Fig. 4 summarizes the knowledge on the *Rp1* gene model available in the maize genetics and genomics database (MaizeGDB, Woodhouse et al., 2021).

### 4.2 RS traits for phenotyping of CR symptoms

Overall, our results illustrate that the RS indices used in this research, in principle, can be used for genomic analyses and can thus replace the labor-intensive VS as a trait. As expected, the methodology can potentially be used to build high-throughput platforms for genomic evaluations.

However, the hypothesis that the objectivity of automated RS data leads to improved data quality was not confirmed. Contrarily, the VS data was of higher quality than any of the RS indices that were used in this work. This view is supported by the lower (broad-sense) heritabilities of RS indices compared to VS (Table 2) for individual population × year combinations, by the reduced across-year correlation of RS traits compared to VS (Table 3), and by the weaker genetic signals detected in linkage analyses (Table 4). The two latter points may in part be a consequence of the reduced heritability, but when considering the heritability of the index traits for populations 2 and 3 in 2020 (Table 2), we see that even when the heritabilities of RS traits are on the same level as the heritability of the VS, the signals observed in linkage analyses are still less pronounced for the RS traits. Therefore, the indices used here may simply not fully align with the symptoms of the disease and may also reflect influences of additional other stresses. A VS assigned by trained staff may distinguish different types of stress better than general vegetation indices built from spectral reflectances, and therefore describe specific disease symptoms better. A further specification of RS indices to the symptoms of the particular disease might reduce the difference in quality between VS and a modified RS index. Also, the quality of the incident light sensor of the camera and the image processing pipeline may influence the quality of the data (as found by Franzini et al., 2019, and Olsson et al., 2021).

Although the overall results point to the VS being of higher quality than the RS indices, please recall that the index G identified the position 2954,643 bp for population 2 in 2019. In light of the knowledge on the position of the *Rp1* cluster described above, this position is likely more precise than position 20,858,205 bp which had the strongest signal for VS in this population × year combination. This could be interpreted as an argument for the index G being more objective, but rather we think that this observation is a result of small variations in data quality, allele frequencies and population size together with both markers being statistically associated with the disease symptoms.

## 5 Conclusion

Overall, our results show that UAV-derived indices can be used for phenotyping resistance to foliar diseases for gene discovery and selection. UAV-based phenotyping may allow processing larger numbers of field plots at a higher rate (high-throughput phenotyping). Larger populations imply a higher number of genetic recombinations in the population which leads to more precise mapping of favorable alleles (Romay et al., 2013). However, our work was not able to support the hypothesis that the objectivity of image data leads to improved data quality. For the example on hand with the respective disease, the volumes, the quality of the VS and the RS indices used, the VS remained the benchmark metric. Whether phenotyping by RS is cost efficient for a breeding program will depend on the respective cost structure, on the number of plots to be evaluated and on how well the chosen RS indices can capture the severity of the symptoms of the respective disease of interest. In this regard, there may also be potential for improvement by refining the index used according to the disease dealt with.

## CRediT authorship contribution statement

**Loladze Alexander:** Conceptualization, Funding acquisition, Investigation, Writing – original draft, Project administration. **Rodrigues JR Francelino A:** Conceptualization, Investigation, Methodology, Writing – original draft. **Petroli Cesar D:** Data curation, Investigation, Writing – original draft. **Muñoz-Zavala Carlos:** Data curation, Formal analysis, Investigation. **Naranjo Sergio:** Resources, Investigation, Data curation. **San Vicente Felix:** Conceptualization, Resources. **Gerard Bruno:** Project administration, Resources, Supervision. **Montesinos-Lopez Osval A:** Data curation, Formal analysis. **Crossa Jose:** Conceptualization, Data curation, Formal analysis, Methodology, Software. **Martini Johannes Wolfgang Robert:** Conceptualization, Data curation, Formal analysis, Investigation, Methodology, Software, Writing – original draft, Writing – review & editing.

## Declaration of Competing Interest

The authors do not have any competing interest that could influence / bias their work.

## Data Availability

The data is available with a link to a CIMMYT repository.
